# Conjugated linoleic acid improves glycemic response, lipid profile, and oxidative stress in obese patients with non-alcoholic fatty liver disease: a randomized controlled clinical trial

**DOI:** 10.3325/cmj.2016.57.331

**Published:** 2016-08

**Authors:** Mehrangiz Ebrahimi-Mameghani, Haleh Jamali, Reza Mahdavi, Farzad Kakaei, Rana Abedi, Bita Kabir-Mamdooh

**Affiliations:** 1 Nutrition Research Center, School of Nutrition, Tabriz University of Medical Sciences, Tabriz, Iran; 2 Student Research Committee, School of Nutrition, Tabriz University of Medical Sciences, Tabriz, Iran; 3 Nutrition Research Center, School of Nutrition, Tabriz University of Medical Sciences, Tabriz, Iran; 4 Department of General Surgery, School of Medicine, Tabriz University of Medical Sciences, Tabriz, Iran; 5 Student Research Committee, School of Nutrition, Tabriz University of Medical Sciences, Tabriz, Iran; 6 Student Research Committee, School of Nutrition, Tabriz University of Medical Sciences, Tabriz, Iran

## Abstract

**Aim:**

To investigate if conjugated linoleic acid supplementation (CLA) affects metabolic factors and oxidative stress in non-alcoholic fatty liver disease (NAFLD).

**Methods:**

The study was a randomized, controlled clinical trial conducted in specialized and subspecialized clinics of Tabriz University of Medical Sciences from January 2014 to March 2015. 38 obese NAFLD patients were randomly allocated into either the intervention group, receiving three 1000 mg softgel of CLA with a weight loss diet and 400 IU vitamin E, or into the control group, receiving only weight loss diet and 400 IU vitamin E for eight weeks. Dietary data and physical activity, as well as anthropometric, body composition, metabolic factors, and oxidative stress were assessed at baseline and at the end of the study.

**Results:**

Weight, body composition, and serum oxidative stress, insulin, and lipid profile significantly improved in both groups, while hemoglobin A1c (HbA1c) levels (*P* = 0.004), total cholesterol to high density lipoprotein ratio (*P* = 0.008), low density lipoprotein to high density lipoprotein ratio (LDL/HDL) (*P* = 0.002), and alanine aminotransferase to aspartate aminotransferase (ALT/AST) ratio (*P* = 0.025) significantly decreased in the intervention group. At the end of the study, fat mass (*P* = 0.001), muscle mass (*P* = 0.023), total body water (*P* = 0.004), HbA1c (*P* < 0.001), triglycerides (*P* = 0.006), LDL/HDL ratio (*P* = 0.027), and ALT/AST ratio (*P* = 0.046) were significantly better in the CLA group than in the control group.

**Conclusion:**

CLA improved insulin resistance, lipid disturbances, oxidative stress, and liver function in NAFLD. Therefore, it could be considered as an effective complementary treatment in NAFLD.

Registration number: IRCT2014020516491N1.

Non-alcoholic fatty liver disease (NAFLD) is the most common chronic liver disease characterized by the accumulation of large droplets of triglycerides within hepatocytes, contributing to more than 5% of liver weight, in the absence of chronic alcohol consumption (1-3). It encompasses a spectrum of pathologic conditions, from simple steatosis to steatohepatitis, cirrhosis, and rarely hepatocellular carcinoma (3). The estimated prevalence of NAFLD in the general population is 20%-30%, increasing to 70%-90% among obese and diabetic patients (3). Its prevalence in Asian countries varies from 9%-40% (4). NAFLD is strongly associated with obesity, type 2 diabetes, dyslipidemia, and hypertension (5) and is thus considered to be the hepatic manifestation of metabolic syndrome (6).

The pathogenesis of NAFLD is not completely understood, but it can be explained by the multi-hit hypothesis, the first hit being steatosis, triggered by insulin resistance, the second hit being oxidative stress and inflammation, resulting in disease progression (7,8), and the third hit being hepatocyte proliferation progenitors impairment (9).

Although there are no specific guidelines for NAFLD treatment, it is recommended to treat the associated factors by weight reduction, glycemic control, and lipid control (7,10). Therefore, functional foods such as bioactive lipids seem to play a role in modulating metabolism and body weight (7). A specific group of 18 carbon poly-unsaturated fatty acids, known as conjugated linoleic acid (CLA) – a mixture of positional and geometric conjugated isomers of linoleic acid (11,12) – has been shown to regulate energy metabolism and is commercially being used as a weight loss supplement (7). Considerable attention has been paid to biological activates of CLA, which act as a potential therapeutic nutrient through their effects on insulin resistance, hyperlipidemia, and controlling oxidative status (13-15).

Recent studies have shown that trans-10, cis-12 CLA isomer reduces body weight and fat accumulation (16) and in some cases increases insulin resistance, impairs blood glucose and lipid profile (15,17-20), and increases oxidative stress and inflammation (21). However, the effects of cis-9, trans-11 and 50:50 isomers are controversial.

Animal studies have shown that CLA supplementation reduces body weight and body fat mass and improves glycemic status and lipid profiles (22-27), but the results in humans are inconsistent (14,22,25-32). CLA has shown no significant effects on lipid profile, fasting blood glucose, insulin resistance, body composition, and body mass index (BMI) among healthy and hyperlipidemic overweight and obese participants (28,29), as well as diabetic patients (14). In diabetic patients, CLA supplementation (3 g/d) showed negative effects on insulin and glucose metabolism and positive effects on serum HDL metabolism (13), triacylglycerol (TAG), and very low density lipoproteins (VLDL), but did not affect any other biochemical parameters (33). However, in another study it improved body composition, serum glucose, and insulin concentrations without having significant effects on lipid profile (34). In addition, CLA supplementation had no significant effects on lipid peroxidation and antioxidant metabolism among healthy volunteers (35), but had beneficial effects on oxidative stress among atherosclerotic patients (36).

Therefore, as NAFLD prevalence is increasing worldwide and studies investigating the effects of CLA supplementation on patients with NAFLD are rare and inconsistent, we aimed to examine whether weight loss diet with and without CLA supplementation had an effect on insulin resistance, lipid profile, and oxidative stress in obese patients with NAFLD.

## Methods

### Participants and methods

This randomized controlled clinical trial was conducted from January 2014 to March 2015. 234 participants referred to the specialized and subspecialized clinics of Tabriz University of Medical Sciences underwent ultrasonography by a single sonographist for determining fatty liver. According to the Saverymuttu method (37), the degree of fatty liver was classified as: “mild,” “moderate,” and “severe.” The study protocol was approved by the Ethics Committee of Tabriz University of Medical Sciences and was registered at the Iranian Registry of Clinical Trials website (IRCT2014020516491N1). Written informed consent was obtained from each participant.

Sample size was estimated to be 19 participants in each group, considering 20% change in mean Quicki index reported by Shadman et al (14) using Pocock formula and confidence level of 95% (α = 0.05) and power of 90% (β = 0.10).

Patients confirmed as NAFLD were assessed based on the inclusion criteria, ie, 20-50 years of age, BMI between 30-40 kg/m^2^, and taking 400 international units (IU) of vitamin E supplement daily. Exclusion criteria were alcohol consumption; pregnancy or lactation, being menopausal and athlete; inflammatory conditions such as infection; hypertension; family history of hyperlipidemia; cardiovascular disease, lung, renal or liver disease; liver transplantation; biliary disease; known autoimmune disease; cancer; burns and injuries during the study; surgery in the last 3 months; use of medications such as antihypertensives, insulin sensitivity enhancers, hepatotoxic drugs, statins, contraceptive pills, and estrogens, as well as vitamin and mineral supplements, and antioxidant supplementation in the last two months. This left 28 and 26 participants in the control and CLA group, respectively (totally 54 patients).

### Study design

**Figure 1:**
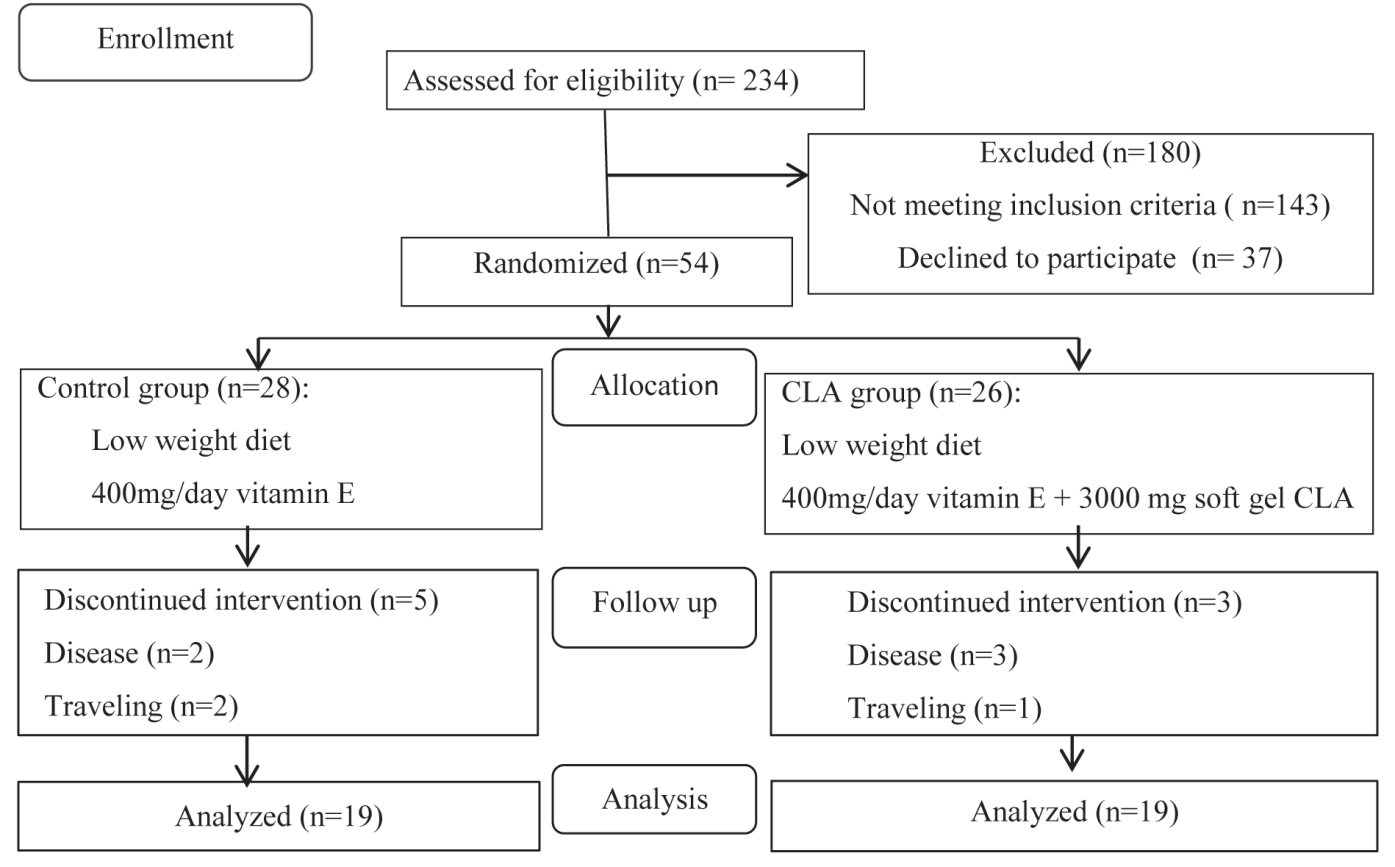
Flowchart of the study.

Demographic characteristics and disease history were obtained. All patients received 400 IU/d vitamin E supplement as routine treatment. The patients were randomly allocated into two groups based on BMI, sex, and NAFLD grade using random block (n = 4): the intervention group receiving CLA 80% soft gel 1000 mg supplied by Nutrifit (Nutricentury, Markham, ON, Canada, containing both cis-9, trans-11, and trans-10, cis-12 type CLAs in equal proportion) three times per day with a weight loss diet meal and the control group receiving weight loss diet only for eight weeks (Figure 1). The CLA dosage used in this study has been shown to be non- toxic and without side effects (38-40). Each patient received their supplements in 4 batches, every 2 weeks. The person who determined allocation sequence for the study and those who assigned participants were blinded. The person who analyzed the data was also blinded.

A food frequency questionnaire (FFQ) (36) for assessing habitual diet was completed at baseline. For weight loss diet, individual energy requirement was estimated based on the current weight using Harris-Benedict formula (41) minus 700 kcal with the 55:30:15% of energy from C:F:P. All participants were asked to maintain their usual diet and lifestyle habits. 16 participants could not adhere to the protocol because of travel, diseases not related to CLA, discontinued intervention because of failure to follow diet recommendation, and irregular consumption of supplements due to the lack of attention. They were excluded from the study.

### Anthropometric and body composition measurement

Weight and height were measured using Seca scale (Hamburg, Germany) and non-stretchable tape to the nearest 100 g and 0.5 cm, respectively, after which BMI was estimated. Waist and hip circumferences were measured after expiration at the midpoint between the lowest rib and the iliac crest and at the widest point between the hip and the buttock to the nearest 0.5 cm, respectively. Waist to hip ratio (WHR) and waist to height ratio (WHtR) were estimated. Body composition was assessed at the beginning and end of the study using body analyzer (Tanita BC-418 Body Composition Analyzer, Arlington Heights, IL, USA)

### Dietary assessment

Mean daily dietary intake was assessed through fulfilling a three 3-day food records at baseline, week 4, and week 8. Home measurements and scales were used to quantify the portion sizes. Energy and macronutrients intakes were analyzed using Nutritionist IV software (ver. 3.5.2, San Bruno, CA, USA).

### Physical activity measurement

Three physical activity questionnaires (1) were completed at baseline, week 4, and the end of intervention and reported as metabolic equivalents (MET) per day.

### Biochemical measurements

10-mL fasting blood samples were taken at the beginning and end of the study. Aliquots of serum were collected in micro tubes and stored at -70^◦^C until analysis. Serum alanine aminotransferase (ALT), aspartate aminotransferase (AST), fasting blood glucose (FBS), insulin, lipid profile (total cholesterol [TC], low density lipoprotein cholesterol [LDL-C], high density lipoprotein cholesterol [HDL-C], and triglycerides [TG]), and oxidative stress indices (total antioxidant capacity [TAC], arylesterase [ARE], and malondialdehyde [MDA]). Liver enzymes were measured using International Federation of Clinical Chemistry and Laboratory Medicine (IFCC) method (Biosystem, Barcelona, Spain kit) using autoanalyzer instrument (Hitachi 911 Depok, Indonesia). Lipid profile and FBS were measured using commercial kits and enzymatic colorimetric method (Parsazmun, Tehran, Iran) with autoanalyzer (Abbot, Model Alycon 300, USA). LDL-C was calculated using the Friedwald formula (42). Insulin was measured using enzyme linked immune assay (ELISA) commercial kit (Monobind Inc. Lake Forest, CA, USA). Insulin resistance (IR) was determined using the homeostasis model assessment (HOMA). HOMA-IR and Quantitive Insulin Sensitivity Check Index (Quicki) scores were calculated using the following formulas (14):

HOMA-IR = fasting blood glucose (mmol/L) × serum insulin (μU/mL) /22.5

Quicki = 1/log fasting blood glucose (mmol/L) + log serum insulin (μU/mL)

Spectrophotometry method with phenylacetate as a substrate was applied to measure serum ARE activity. TAC was measured using spectrophotometry method with Randox TAS kit (radical ABTS; Randox Laboratories, Antrim, United Kingdom). However, spectrophotometry technique for MDA assessment was based on its reaction with thiobarbituric acid.

### Statistical analysis

All statistical analyses were performed using SPSS software (ver. 16.0, SPSS Inc., Chicago, IL, USA). The normality of distribution of continuous variables was tested using Kolmogorov-Smirnov test. Data for normally distributed variables are shown as mean ± standard deviation. Independent *t* test was used to compare the variable means between the two groups and analysis of covariance (ANCOVA) was applied for adjusting the covariates. To compare the change in the studied variables over the study period in each group, paired *t* test and Wilcoxon rank *t* test were used. A repeated measure analysis was also applied to assess the changes in dietary intakes and physical activity over the study period. *P*-<0.050 was considered as significant.

## Results

38 of 54 patients (19 in each group) completed the study (Figure 1). At baseline, there were no significant differences between the groups in age (36.74 ± 6.87 years and 38.58 ± 8.24 years in intervention and control group, respectively), sex, marital status, education level, and severity of fatty liver disease (Table 1). The control group had significantly greater mean weight, waist and hip circumferences than CLA group (*P* = 0.031, *P* = 0.030, and *P* = 0.015, respectively), Thus, these factors were considered as covariates in the analysis (Table 2). At the end of the study, anthropometric measurements decreased significantly in both groups, but there were no significant differences between the groups. Fat mass, muscle mass, and total body water improved significantly after the study in the CLA group compared to the control group (*P* = 0.001, *P* = 0.023, and *P* = 0.004, respectively).

**Table 1:** Baseline characteristics of the group treated with conjugated linoleic acid and controls

Characteristic	Control (n = 19) (%)	Conjugated linoleic acid (n = 19) (%)	P*
Female	84.2	89.5	0.999
Single	89.5	100	0.486
Educational level			
before high school and high school	57.9	57.9	0.999
university degree	42.1	42.1	
Severity of fatty liver			
mild	78.9	68.4	0.461
moderate and severe	21.1	31.6	

*χ^2^ test.

**Table 2:** Anthropometric measurements and body composition before and after conjugated linoleic acid supplementation (CI – confidence interval, MD – mean difference)

Variable	Control (n = 19, mean ± standard deviation)	Conjugated linoleic acid (n = 19, mean ± standard deviation)	*P*
Height (cm)	159.24 ± 6.67	158.81 ± 8.93	0.867^†^
Bone	2.726 ± 0.378	2.942 ± 0.373	0.105^†^
Weight (kg)			
before	89.36 ± 9.34	82.10 ± 10.60	0.031^†^
after	84.84 ± 9.56	77.30 ± 10.45	0.559^‡^
MD (CI 95%)	4.5(3.71 to 5.28)	4.80(4.10 to 5.50)	
P*	**<0.001**	**<0.001**	
Body mass index (kg/m^2^)			
before	35.27 ± 3.46	32.72 ± 4.63	0.064^†^
after	33.50 ± 3.63	30.80 ± 4.45	0.460^‡^
MD (CI 95%)	1.77(1.46 to 2.07)	1.97(1.26 to 2.22)	
P*	**<0.001**	**<0.001**	
Waist circumference(cm)			
before	109.28 ± 9.92	103.18 ± 6.34	0.030^†^
after	103.70 ± 10.34	97.34 ± 7.01	0.952^‡^
MD (CI 95%)	5.58(4.63 to 6.53)	5.84(4.72 to 6.96)	
P*	**<0.001**	**<0.001**	
Hip circumference(cm)			
before	119.73 ± 7.89	113.68 ± 6.75	0.015^†^
after	116.11 ± 8.19	109.08 ± 7.09	0.342^‡^
MD (CI 95%)	4.60(3.45 to 5.57)	3.63 (2.69 to 5.56)	
P*	**<0.001**	**<0.001**	
Waist to hip ratio			
before	0.91 ± 0.047	0.90 ± 0.047	0.804^†^
after	0.89 ± 0.066	0.89 ± 0.054	0.530^‡^
MD (CI 95%)	0.02(0.01 to 0.021)	0.01(0.00 to 0.02)	
P*	**<0.001**	**<0.001**	
Waist to height ratio			
before	0.68 ± 0.067	0.65 ± 0.053	0.080^†^
after	0.65 ± 0.069	0.61 ± 0.054	0.778^‡^
MD (CI 95%)	0.03(0.02 to 0.04)	0.03 (0.02 to 0.04)	
P*	**<0.001**	**<0.001**	
Fat mass (%)			
before	46.71 ± 6.35	46.05 ± 5.71	0.737^†^
after	45.55 ± 6.69	41.43 ± 6.46	**0.001^‡^**
MD (CI 95%)	1.16(0.39 to 1.93)	4.61(2.81 to 6.41)	
P*	**0.005**	**<0.001**	
Muscle mass (%)			
before	32.44 ± 4.36	33.92 ± 2.57	0.210^†^
after	33.02 ± 4.34	35.55 ± 2.79	**0.023^‡^**
MD (CI 95%)	-0.63(-0.82 to -0.44)	-1.63(-2.39 to -0.88)	
P*	**<0.001**	**<0.001**	
Total body water			
before	36.31 ± 4.35	36.04 ± 3.90	0.846^†^
after	38.14 ± 4.59	38.14 ± 4.41	**0.004^‡^**
MD (CI 95%)	-0.45(-1.00 to 0.09)	-0.094(-3.02 to -1.161)	
P*	0.102	**<0.001**	

*Paired t- test.

†Independent *t* test.

^‡^Analysis of covariance (ANCOVA) adjusted for baseline values and energy intake.

There were no differences in energy and macronutrient intakes between the two groups at baseline, week 4, and week 8, except for fat intake, which was significantly higher at week 8 in the CLA group (*P* = 0.019). Significant change in total energy intake in the control group was considered as a confounder in advanced analysis. Repeated measure analysis found no significant changes in physical activity (expressed as MET/d) over the study period (Table 3).

**Table 3:** Daily total energy and macronutrient intakes and physical activity

Variable	Control (n = 19, mean ± standard deviation)	Conjugated linoleic acid (n = 19, mean ± standard deviation)	P*
Energy (kcal)			
week 0	1272.71 ± 292.33	1120.68 ± 306.25	0.126
week 4	1124.09 ± 334.92	1154.96 ± 252.81	0.757
week 8	1157.18 ± 340.92	1171.45 ± 325.60	0.897
*P^†^*	**0.020**	0.921	
Carbohydrate (g)			
week 0	192.73 ± 56.51	161.67 ± 56.57	0.099
week 4	176.57 ± 59.45	165.09 ± 35.30	0.416
week 8	182.85 ± 69.23	163.72 ± 42.53	0.321
*P^†^*	0.441	0.833	
Protein (g)			
week 0	52.70 ± 14.03	49.96 ± 13.92	0.510
week 4	51.44 ± 14.21	49.77 ± 12.20	0.708
week 8	48.35 ± 10.40	48.19 ± 12.55	0.887
*P^†^*	0.298	0.808	
Fat (g)			
week 0	30.71 ± 8.09	31.90 ± 9.41	0.680
week 4	28.20 ± 12.19	35.83 ± 13.26	0.081
week 8	26.88 ± 7.98	34.90 ± 11.67	**0.019**
*P^†^*	0.213	0.435	
Physical activity (metabolic equivalents /d)			
week 0	36.41 ± 4.20	38.27 ± 2.69	0.117
week 4	36.06 ± 4.12	38.69 ± 2.61	0.027
week 8	36.75 ± 3.76	38.28 ± 2.44	0.148
*P^†^*	0.203	0.439	

*Independent *t* test.

†Repeated measure analyses.

At baseline, the groups had similar glycemic index and liver enzyme, while CLA group had significantly higher total cholesterol (*P* = 0.042). At the end of the study, fasting glucose concentration non-significantly decreased in CLA group. HbA1c significantly decreased in the CLA group during the study (*P* = 0.004) and after the intervention it was lower than in the control group (*P* < 0.001). Insulin significantly increased during the study in both groups (*P* = 0.024 and *P* = 0.020 in the control and CLA group, respectively). HOMA-IR score significantly increased at week 8 compared to baseline in the control group (*P* < 0.001), while it decreased non-significantly in the CLA group. Quicki index increased non-significantly in both groups. Also, total cholesterol levels, triglycerides, and LDL significantly decreased in both groups, and TC/LDL and LDL/HDL ratios significantly decreased only in the CLA group (*P* = 0.008 and *P* = 0.002, respectively). Also, TG and LDL/HDL ratio significantly decreased in the CLA group compared to the control group (*P* = 0.006 and 0.027, respectively). ALT/AST ratio significantly decreased in the CLA group (*P* = 0.025). While this ratio non-significantly increased in the control group, the reduction in ALT/AST ratio in CLA group was significantly higher than in the control group (*P* = 0.046) (Table 4).

**Table 4:** Metabolic variables before and after conjugated linoleic acid supplementation (CI – confidence interval; MD – mean difference)

Variable	Control (n = 19, mean ± standard deviation)	Conjugated linoleic acid (n = 19, mean ± standard deviation)	*P*
Fasting blood glucose (mg/dL)			
before	96.16 ± 10.43	102.26 ± 18.05	0.210^†^
after	98.47 ± 11.29	98.68 ± 10.26	0.683^‡^
MD (CI 95%)	1.16 (0.39 to 1.93)	4.61 (2.81 to 6.41)	
P*	0.074	0.392	
Hemoglobin A1c (%)			
before	4.57 ± 0.08	4.49 ± 0.77	0.774^†^
after	4.74 ± 0.88	3.99 ± 0.63	**<0.001^‡^**
MD (CI 95%)	-0.17 (-0.36 to 0.02)	0.50 (0.17 to 0.83)	
P*	0.077	**0.004**	
Insulin (μIU/mL)			
before	13.87 ± 7.34	13.37 ± 4.04	0.797^†^
after	14.55 ± .7.45	13.89 ± 4.25	0.423^‡^
MD (CI95%)	-0.67 (-1.25 to -1.00)	-0.51 (-0.94 to -0.09)	
P*	**0.024**	**0.020**	
Homeostatic model assessment-insulin resistance			
before	3.26 ± 1.77	3.4 ± 1.33	0.795^†^
after	3.50 ± 1.83	3.39 ± 1.12	0.178^‡^
MD (CI 95%)	-0.23 (-0.03 to -0.12)	0.006 (-0.26 to 0.27)	
P*	**<0.001**	0.096	
Quantitative insulin sensitivity check index			
before	1.57 ± 0.29	1.60 ± 0.15	0.669^†^
after	1.59 ± 0.26	1.62 ± 0.14	0.495^‡^
MD (CI 95%)	-0.02 (-0.06 to 0.001)	-0.01 (-0.09 to 0.00)	
P*	0.080	0.053	
Cholesterol(mg/dL)			
before	188.74 ± 27.76	213.11 ± 41.66	0.042^†^
after	176.95 ± 24.62	203.37 ± 39.51	0.179^‡^
MD(CI 95%)	11.78 (6.26 to 17.31)	9.737 (1.75 to 17.21)	
P*	**<0.001**	**0.020**	
Triglyceride(mg/dL)			
before	164.05 ± 92.27	149.42 ± 63.60	0.573^†^
after	143.95 ± 81.69	110.15 ± 48.46	**0.006^‡^**
MD (CI 95%)	20.10 (7.098 to 33.11)	39.26 (27.54 to 50.99)	
P*	**0.004**	**<0.001**	
Low density lipoprotein (mg/dL)			
before	25.23 ± 26.73	138.53 ± 33.13	0.182^†^
after	115.58 ± .24.72	122.80 ± 29.53	0.616^‡^
MD (CI 95%)	9.65 (5.02 to 14.28)	15.72 (6.16 to 25.28)	
P*	**<0.001**	**0.003**	
High density lipoprotein (mg/dL)			
before	45.95 ± 7.46	49.63 ± 15.41	0.354^†^
after	44.89 ± 9.86	51.95 ± 16.90	0.114^‡^
MD (CI 95%)	1.05 (-1.20 to 3.31)	-2.31 (-6.87 to 2.24)	
P*	0.34	0.300	
Total cholesterol/high density lipoprotein			
before	4.19 ± 0.77	4.72 ± 1.88	0.259^†^
after	4.09 ± 0.9	4.26 ± 1.52	0.150^‡^
MD (CI 95%)	0.09 (-0.06 to 0.24)	0.46 (0.13 to 0.79)	
P*	0.277	**0.008**	
Low density lipoprotein /high density lipoprotein			
before	2.79 ± 0.68	3.08 ± 1.28	0.380^†^
after	2.70 ± 0.84	2.56 ± 0.93	**0.027^‡^**
MD (CI 95%)	0.08(-0.07 to 0.23)	0.52 (0.22 to 0.83)	
P*	0.270	**0.002**	
Aspartate aminotransferase (Iu/L)			
before	18.26 ± 3.94	19.89 ± 17.36	0.405^†^
after	18.05 ± 3.14	17.33 ± 4.21	0.375^‡^
MD (CI 95%)	0.21 (-1.50 to 1.93)	2.55 (-0.41 to 5.52)	
P*	0.800	0.087	
Alanine aminotransferase (Iu/L)			
before	22.37 ± 9.04	24.82 ± 23.01	0.798^†^
after	31.74 ± 28.24	17.83 ± 10.40	0.086^‡^
MD (CI 95%)	-8.36 (-22.04 to 5.30)	7 (-1.16 to 15.16)	
P*	0.81	0.215	
Alanine aminotransferase/ aspartate aminotransferase			
before	1.24 ± 0.31	1.12 ± 0.44	0.340^†^
after	1.81 ± 1.95	0.98 ± 0.39	**0.046^||^**
*P*^§^	0.159	**0.025**	
Total antioxidant capacity (mmol/L)			
before	1.36 ± 0.31	1.21 ± 0.34	0.182^†^
after	1.58 ± 0.38	1.32 ± 0.42	0.285^‡^
MD (CI 95%)	-0.22 (-0.36 to -0.07)	-0.0/1 (-0.2 to 0.01)	
P*	**0.005**	**0.028**	
Aryl esterase (u/L)			
before	111.0 ± 29.85	118.89 ± 28.41	0.409^†^
after	124.26 ± 30.99	133.68 ± 21.54	0.694^‡^
MD (CI 95%)	-13.26 (-20.05 to -6.47)	-14.78 (-23.05 to -6.53)	
P*	**0.001**	**0.001**	
Malondialdehyde (mmol/mL)			
before	2.57 ± 0.61	2.87 ± 1.10	0.314^†^
after	2.31 ± 0.57	2.38 ± 0.59	0.437^‡^
MD (CI 95%)	021 (0.15 to 0.37)	0.49 (0.17 to 0.80)	
P*	**<0.001**	**0.004**	

*Paired t- test.

†Independent *t* test.

‡Analysis of covariance (ANCOVA) adjusted for baseline values, energy intake, weight circumference and hip circumference.

§Wilcoxon rank *t* test.

||Mann-Whitney u test.

## Discussion

To our knowledge, this is the first study investigating the effects of CLA in combination with vitamin E and low-calorie diet in patients with NAFLD disease. We found that CLA supplementation improved body composition, lipid profile, oxidative stress, liver function, and serum HbA1c.

In our study, CLA group had decreased insulin resistance assessed by HOMA-IR, which might be attributed to energy expenditure, increasing peroxisome proliferators-activated receptor (PPARγ) expression – acting as a ligand for this receptor (22) and adiponectin production (43). Our findings were similar to a study on sedentary women with metabolic syndrome (44), healthy women (34), and diabetic patients (13). However, in most studies in humans, CLA supplementation had no effect on glycemic status and lipid profile (14,25,29,31,32). In the present study, CLA supplementation improved lipid profile and significantly reduced serum TG levels (12.25%) and LDL/HDL ratio (16.88%), which was shown to be the best single predictor of cardiovascular disease (45). Therefore, the effect of CLA supplementation on LDL/HDL cholesterol in NAFLD patients may be of clinical benefit. Maloney et al (13) showed that a similar dose of CLA in a similar study period as in our study significantly reduced LDL/HDL cholesterol ratio among type 2 diabetic patients. Another study showed that CLA supplementation significantly improved plasma TG levels in normo-lipidemic participants (33), while no changes in serum total cholesterol, HDL, LDL, and triglyceride level were found among healthy women (34). In contrast, 5.5 g/d CLA for 16 weeks increased serum TG and TC/HDL ratio and decreased HDL-C among postmenopausal women (46). The inconsistency in results may be due to differences in study populations, CLA dose, and the intervention period. The hypotriacylglycerol effect of CLA might be explained by the fact that CLAs are potent agonists of PPARs, including PPARα, which is the key transcription factor regulating hepatic lipid metabolism, and PPARγ, which regulates the expression of the genes determining adipogenesis, lipid metabolism, and insulin sensitivity. Some other proposed mechanisms affect peroxisome proliferators-sterol regulatory element-binding proteins (SREBPs), and stearoyl-CoA desaturase (SCD). SREBP1c isoforms regulate fatty acid and TG synthesis (47).

The present study found non-significant reductions in ALT serum levels (28.16%), however the change in ALT/AST ratio (12.5%) was significant. Although there is a limited number of studies examining the effect of CLA mix on liver function, Nagoa et al (43) found that 8-week diet containing 1% CLA significantly decreased serum AST and ALT levels in obese, diabetic Zucker rats. Another study also found that CLA consumption significantly reduced serum ALT concentration in Zucker rats (27). On the other hand, a study in healthy non-obese sedentary women, similarly to our study, failed to show any significant difference in serum ALT levels (48). The effect of CLA on liver function could be due to the enhancement of adiponectin production (43).

ARE is synthesized by the liver and hydrolyzes organophosphate compounds in mammals (49). In our study, serum ARE levels increased significantly in both groups, but no significant difference was shown between the two groups. There is a limited number of studies examining the effects of CLA supplementation on serum ARE levels. Ariyaeian et al (47) reported no significant difference in serum ARE level between patients with rheumatoid arthritis who received supplementation with 2gr CLA + 400 mg vitamin E and those who received corn oil for three months. In the present study, serum MDA levels decreased significantly and TAC increased significantly without any differences between the groups. The studies on Sprague-Dawley rats and mice (15,50) showed that CLA significantly reduced MDA levels. In addition, Aliasghari et al (36) reported a significant reduction of MDA after CLA supplementation in atherosclerosis. The effect of CLA on oxidative stress improvement might be explained by its antioxidant activity (51), induction of the activity of antioxidant enzymes (52), and decreasing lipid peroxidation (53).

This study has several limitations. It was not possible to estimate dietary intake and serum CLA concentration. We also had a relatively small sample size, short follow-up period, and did not use placebo in the control group. Therefore, further long-term placebo-controlled clinical trials are required to study the effect CLA supplementation. Due to insufficient information regarding molecular mechanisms of CLA in humans, human cell culture studies are also recommended.

## 

Accepted: May 2, 2016
